# Recent Progress and Development of G-Quadruplex-Based Luminescent Assays for Ochratoxin A Detection

**DOI:** 10.3389/fchem.2020.00767

**Published:** 2020-09-01

**Authors:** Sang-Cuo Nao, Ke-Jia Wu, Wanhe Wang, Chung-Hang Leung, Dik-Lung Ma

**Affiliations:** ^1^State Key Laboratory of Quality Research in Chinese Medicine, Institute of Chinese Medical Sciences, University of Macau, Taipa, China; ^2^Department of Chemistry, Hong Kong Baptist University, Kowloon Tong, China

**Keywords:** ochratoxin A, G-quadruplex, food safety, nucleic acid detection, aptamer

## Abstract

Ochratoxin A (OTA) is a mycotoxin that is widespread throughout the world. It contaminates foods such as vegetables, fruits, and rice. It harms human health and has potential carcinogenic effects. The G-quadruplex (G4) is a tetraplexed DNA structure generated from guanine-rich DNA that has found emerging use in aptamer-based sensing systems. This review outlines the status of OTA contamination and conventional detection methods for OTA. Various G4-based methods to detect OTA developed in recent years are summarized along with their advantages and disadvantages compared to existing approaches.

## Introduction

Mycotoxins are a large class of secondary metabolites with diverse chemical structures and biological effects. They contaminate foodstuffs throughout the world and may cause illness and even death (Richard, 2007). Ochratoxins are a class of mycotoxins that consists of three members: A, B, and C. Ochratoxin A (OTA), produced by *Aspergillus ochraceus*, is the most common and also the most poisonous of the ochratoxins (Umar et al., 2012). It is recognized as one of the five most important agricultural mycotoxins (Shephard, 2008). OTA is found in everyday foods such as cocoa, cereals, coffee, nuts, spices, meats, eggs, beer, wine, dairy products, and fruits (Yang et al., 2018). OTA is highly stable and resists conditions of acid and heat. OTA persisted after high temperature at 180°C heat, and only degraded after minor extent of heat impact or above 180°C (Raters and Matissek, 2008). Therefore, it is challenging to eliminate OTA once foods are contaminated.

OTA inhibits protein synthesis as well as nucleic acid synthesis (Creppy et al., 1980). Many studies have shown that OTA is harmful to human health mainly in the form of nephrotoxicity, hepatotoxicity, neurotoxicity, teratogenicity, carcinogenicity, and immunotoxicity (El Khoury and Atoui, 2010). The International Agency for Research on Cancer (IARC) has classified OTA as a possible human carcinogen (Abarca et al., 2001). The European Union has specified limits for OTA of 5 ppb in cereals and 2 and 5 ppb in wine and coffee, respectively. Therefore, detection technologies are required to meet regulatory requirements and reduce health risks from OTA contamination.

In order to detect trace OTA in food extracts, a number of analysis methods have been reported, including mass spectrometry (MS) (Huang et al., 2014; Roland et al., 2014), high performance liquid chromatography (HPLC) (Blank and Blind, 2005; Pussemier et al., 2006), and antibody-based techniques (Venkataramana et al., 2015). However, these methods are generally time-consuming, labor-intensive, and require high-tech equipment and trained operators. Therefore, sensitive, accurate, fast, and portable detection methods are urgently required for detecting OTA in food samples.

Aptamers are oligonucleotides that have high affinity for specific targets. Aptamer-based detection methods has emerged as platforms for various bioanalytical application, including for OTA detection. These assays are mainly coupled with fluorescence signal analysis (McKeague et al., 2014; Wang B. et al., 2016), electrochemical analysis (Mishra et al., 2016; Wang Q. et al., 2016), colorimetric analysis (Lee et al., 2014; Wang B. et al., 2016; Yin et al., 2017).

The G-quadruplex (G4), a four-stranded nucleic acid structure, is formed from guanine-rich sequences. Guanine tetrads are stabilized through Hoogsteen hydrogen bonding as well as certain cations or ligands (Simonsson, 2001). G4s are ubiquitous in organisms and are involved in various cellular events including recombination, gene replication and, telomere extension, and gene expression regulation (Huppert and Balasubramanian, 2005). G4s are implicated in cell aging, apoptosis, and immune system function, while defects in G4 biology are related to occurrence of tumors and genetic diseases (Lipps and Rhodes, 2009; Wu and Brosh, 2010). Some ligands such as telomestatin, tetra-(N-methyl-4-pyridyl) porphyrin (TMPyP4), BRACO-19, and RHPS4, can specifically recognize and bind to G4s.

The ability of certain small molecules and toxic substances to bind to G4s has stimulated the development of G4-based detection platforms (Ma et al., 2017). Molecules that can be detected by G4-based assays include amino acids, sugar, adenosine, food toxins, and peptides (Lim et al., 2009). In previous research by our group, we have successfully developed G4-based assays for arsenic ion (Lin et al., 2017), ribonuclease H (Lu et al., 2016d), nickel endonuclease Nb.BsmI (Dong et al., 2017), Siglec-5 (Lin et al., 2016b), specific-gene-deletion (Wang et al., 2017), thymine DNA glycosylase (Lin et al., 2016a), platelet-derived growth factor-BB (Lu et al., 2016a), lysozyme (Lu et al., 2016c), transcription factor HIF-1α (Lu et al., 2016b), insulin content detection (Wang et al., 2015).

In recent years, strategies for detecting OTA using G4s have also emerged. These methods have a common advantage, namely specificity and speed. In this review, we will first introduce current status of OTA detection, and then focus on new strategies for G4-based detection as applied to OTA.

## Ochratoxin A Detection

OTA can cause kidney atrophy, fetal malformation, abortion, and death in test animals (Al-Anati and Petzinger, 2006), and is highly carcinogenic. Researchers have developed different strategies for detecting OTA in foods, such as quartz crystal microbalances (QCM), electrochemical impedance spectroscopy (EIS), and surface plasmon resonance (SPR).

Most existing detection platforms for OTA can be divided into chemical or biological methods. These will be discussed individual in the sections below.

### Chemical Detection Methods for OTA

**Figure 1:**
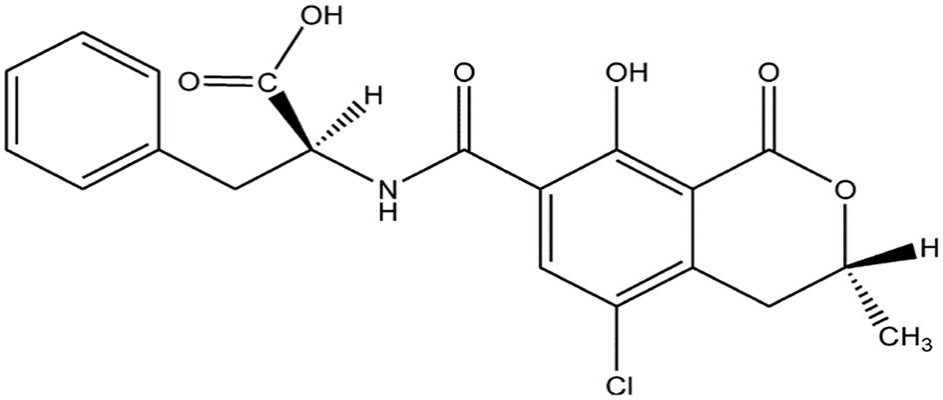
Chemical structure of OTA.

OTA is an organic acid, soluble in sodium bicarbonate and various organic solvents (Figure 1). Qualitative or quantitative detection methods for OTA based on its chemical properties have been described, including thin layer chromatography (TLC), HPLC, MS, high performance thin layer chromatography (HP-TLC), reverse-phase thin-layer chromatography (RP-TLC), and gas chromatography (GC).

TLC equipment is simple and easy to operate, but there are many factors that affect reproducibility and precision. Using TLC, the OTA content in wine products was detected within 2 μg/L, which is the legal limit for OTA in wine stipulated by the European Commission (Teixeira et al., 2011). RP-TLC can be used confirm results obtained via normal phase TLC (Valenta, 1998). Frohlich et al. (1988) detected OTA by using RP-TLC, demonstrating a proportional response with high recovery values recovery (94%). Recently, TLC methods have been combined with immunoaffinity to enhance detection (as discussed below). Although TLC is cheap and simple, it has the drawback of lack of automation.

HPLC has been used to detect OTA, with one study reporting a limit of detection (LOD) of 0.05 ng/mL which was 100 times more sensitive than HPTLC (Kupski and Badiale-Furlong, 2015). Ultra-high-pressure liquid chromatography coupled to tandem mass spectrometry (UHPLC–MS/MS) was used to detect OTA in egg extract with an LOD of 10 μg/kg (Frenich et al., 2011). HPLC coupled with fluorescence detection has also been reported for OTA determination at 0.001 μg/L in food as well as human blood or serum (Filek and Lindner, 1996). As a high-precision instrument, HPLC has the advantages of high detection limit, sensitivity, reliability, and amenability to automation.

GC combined with mass detection has been reported to be potentially useful as a confirmatory tool for samples with OTA ≥ 0.1 μg/L (Soleas et al., 2001). Other detection platforms based on GC-MS have also obtained good results (Jiao et al., 1992; Zhang X. et al., 2019).

For the most part, chromatographic assays tend to be expensive, time-consuming, and require technical instrumentation and personnel. This has stimulated the development of biological detection methods for OTA, which are discussed in the section below.

### Biological Detection Methods for OTA

A number of biological detection methods have been reported for OTA. Immunoaffinity column thin layer chromatography (IM-TLC) has been utilized for detecting OTA in everyday foods. IM-TLC was used to detect OTA with an LOD of 0.5 mg/kg (Santos and Vargas, 2002). In another study, Immunoaffinity column high-performance thin layer chromatography (IM-HPTLC) has also been used to detect OTA with limits of detection <0.28 μg/kg (Chompurat et al., 2007). More recently, an LOD of around 0.4 ng/mL was reported with multitime-regenerated IM (Liu X. et al., 2018). Meanwhile, Yuan et al. (2009) reported a rapid and highly sensitive competitive immunoassay coupled to surface plasmon resonance (IM-SPR) for OTA quantification in cereals and beverage. By applying gold nanoparticles for signal enhancement, the limit of detection was improved from 1.5 to 0.042 ng/ml.

A competitive peptide enzyme-linked immunosorbent assay (ELISA) was developed to detect OTA using the biotinylated peptide “GMVQTIF-GGGSK-biotin” (Zou et al., 2016). The platform exhibited linearity for OTA detection in the range of 0.005–0.2 ng/mL with an LOD of 0.001 ng/mL. However, a drawback of ELISA is that it requires expensive enzyme-labeled reagents, and the procedure is also time-consuming and reagent-intensive. Therefore, researchers have sought methods to enhance the efficiency of ELISA For example, combining nanotechnology or different detection methods (such as fluorescence, colorimetry, and chemiluminescence) with conventional ELISA technology has been explored (Morrissey, 2003).

## Emerging Aptamer Detection Technologies: G-Quadruplex Detection Platform

### Characteristics of G-Quadruplexes

**Figure 2:**
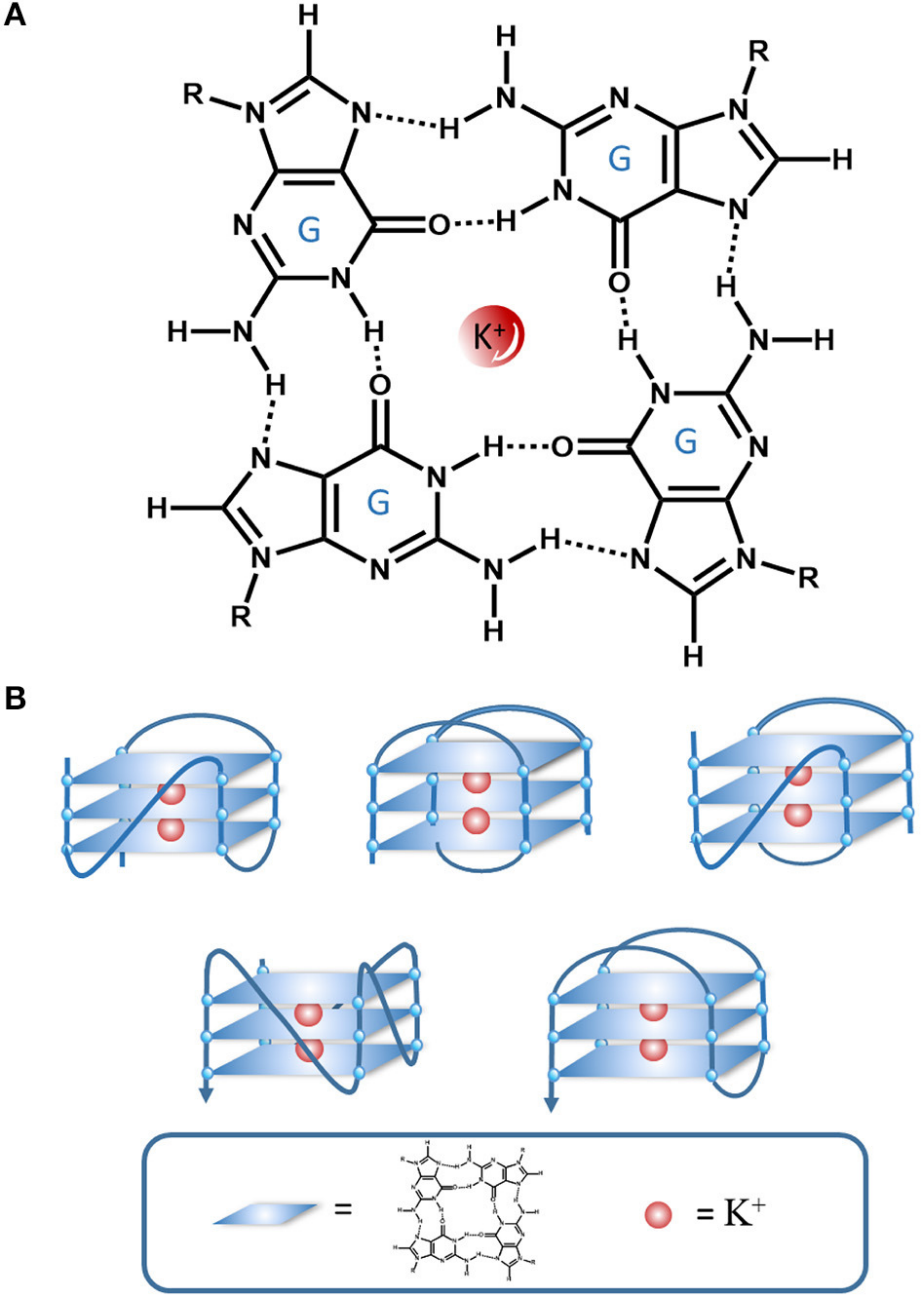
An illustration of the interactions in a G-quartet. **(A)** G-tetrad stabilized interactions by Hoogsten hydrogen bonding and K^+^, **(B)** Various G4 topologies.

The G4 is a tetraplexed nucleic acid secondary structure generated from guanine (G)-rich sequences. The core unit of a G4 is the G-tetrad, which consists of four guanine bases stabilized by Hoogsteen hydrogen bonding (Figure 2A). Multiple G-tetrads can interact through a π-π stacking interactions to form various G4 topologies, such as parallel or anti-parallel structures with different groove and loop regions depending on the nucleic acid sequence (Figure 2B) (Burge et al., 2006). The G4 can be further stabilized by monocations in the central channel, especially sodium or potassium ions (Monchaud and Teulade-Fichou, 2008; Georgiades et al., 2010).

The consensus sequence of (G ≥ 3NxG ≥ 3Nx-G ≥ 3NxG ≥ 3) has been reported to be associated with G4 formation in the genome and transcriptome (Huppert and Balasubramanian, 2005). G4s can participate in various biological processes in living organisms, including gene replication, expression regulation, recombination, apoptosis, cell aging, and autoimmunity (Huppert and Balasubramanian, 2005). Current research focuses on the role of G4s in disease, such as cancer or other genetic diseases (Lipps and Rhodes, 2009; Wu and Brosh, 2010; Kwok and Merrick, 2017).

G4s are especially enriched in the promoter region of genes including those with clinical significance (e.g., *C-MYC, BCL-2, C-KIT, K-RAS*) (Siddiqui-Jain et al., 2002; De Armond et al., 2005; Cogoi and Xodo, 2006). Hence, G4s have arisen as potential targets for chemotherapy (De Cian et al., 2008; Huppert, 2008).

More recently, non-canonical G4s have been discovered, such as quadruplex–duplex constructs (Varizhuk et al., 2017), triplex-quadruplex structures (Bing et al., 2017), as well as a unique type of G4 that contains a G-vacancy and is stabilized by guanine derivatives (Li et al., 2015).

### G-Quadruplex Ligands and Probes

The role of G4s in biology has stimulated the design of small molecules to target these structures. G4 ligands should not only show high affinity for G4s, but also specificity for G4 over other DNA forms, particularly duplex DNA. Ligands with aromatic motifs can interact with G4s through π-π stacking interactions with the terminal G-tetrads, or insertion between G-tetrads. Meanwhile, ligands can also interact with the grooves or loop regions of G4s via electrostatic interactions with the negatively-charged phosphate backbone. Many ligands (e.g., TMPyP4, BRACO-19, RHPS4) have been reported to specifically recognize and bind G4s.

G4 ligands with emissive properties can function as luminescent G4 probes (Ma et al., 2016). Generally, luminescent G4 probes can be broadly classified into two types: organic dyes or metal complexes (Ma et al., 2015). To date, a large number of G4-selective probes have been reported in the literature, including 3,3′-diethyloxydicarbonocyanine (DODC), thiazole orange (TO), thioflavin T (TFT), crystal violet (CV), TMPyP4, and cationic porphyrin anthraquinone diads (CPAD). Meanwhile, metal complexes based on ruthenium(II), iridium(III), platinum(III), and zinc(II) have also been developed as G4 probes.

## G-Quadruplex-Based Detection Methods for OTA

Aptamers are nucleic acid sequences that have been selected for high affinity binding to cognate targets (Ellington and Szostak, 1992). Interestingly, certain aptamers adopt G4 structures upon binding to their targets, including the OTA aptamer. This has stimulated the development of G4-based detection methods for OTA. These methods have the advantages of sensitivity, high selectivity, and low cost compared with traditional methods.

### Labeled Luminescence OTA Detection Methods

Luminescence is a highly popular technique for the determination of chemical or biological substances. Compared to other approaches, luminescence detection has the advantages of sensitivity and simplicity. Aptamer labeling with a fluorescent group is a commonly used strategy for luminescence detection. When the aptamer recognizes its target, it undergoes a conformational change that results in a change in the emission intensity of the fluorescence label.

**Figure 3:**
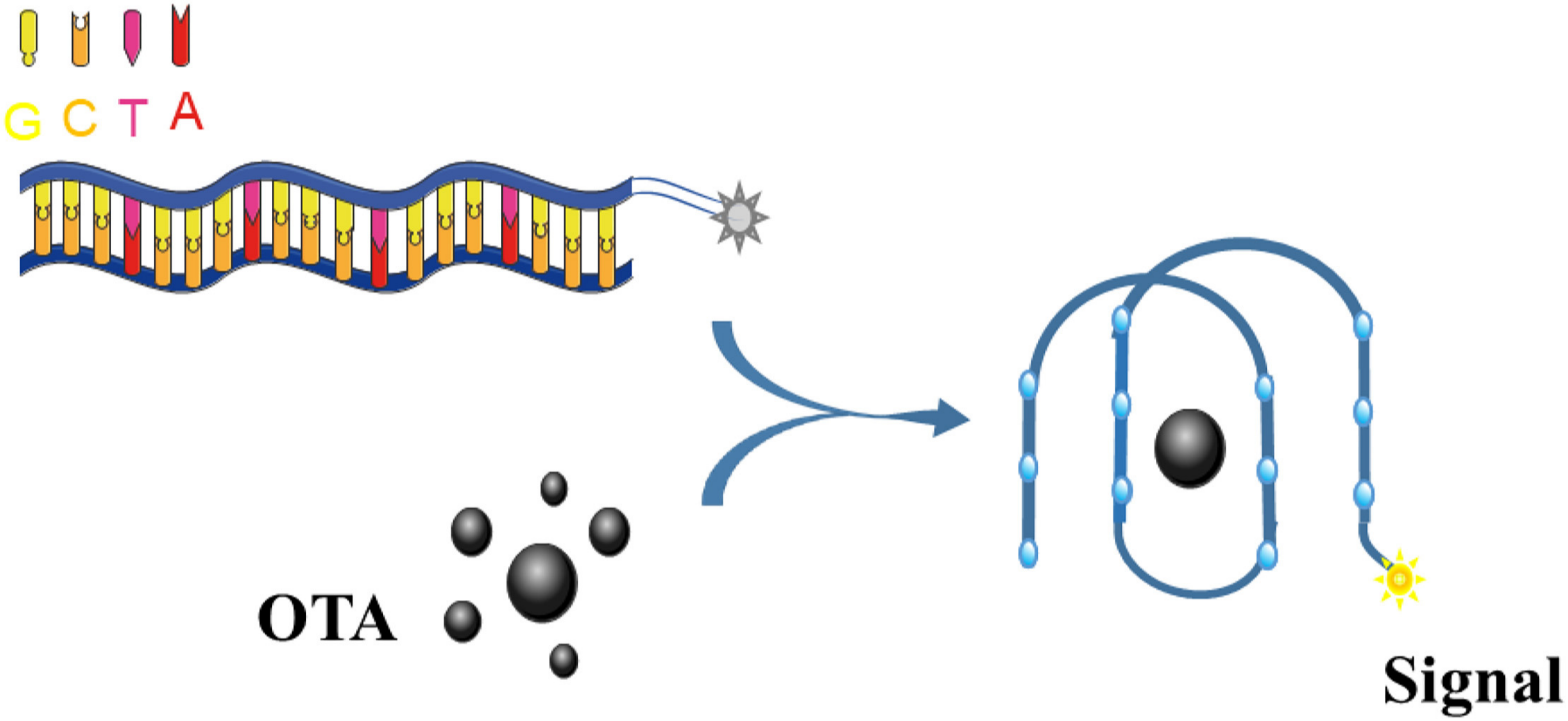
An illustration of the OTA detection process based on a fluorescently-labeled G4 aptamer.

Upon binding to OTA, the OTA aptamer adopts a G4 structure (Figure 3). Thus, this conformational change can be exploited by labeling the aptamer with a fluorescent dye, thus allowing the binding event to be recognized with a emission response.

Guo's group reported a strategy to detect OTA by using a FAM (carboxyfluorescein)-modified aptamer. In their design, the FAM-modified aptamer cannot be quenched by single-walled carbon nanohorn sensors (SWCNHs) after binding to OTA due to the formation of G4 (Lv et al., 2016). In another system, a signal-amplification assay using an Mg^2+^-dependent DNAzyme achieved a LOD for OTA of 140 pmol/L (Guo et al., 2013).

In 2014, Duan's group reported a nano-graphite-aptamer hybrid with the action of DNase I for the amplified monitoring of OTA (Wei et al., 2015). Meanwhile, a one-step chemiluminescent aptasensor was developed by Lee et al., using chemically initiated electron exchange luminescence (CIEEL) between a 1,1′-oxalyldiimidazole chemiluminescence (ODI-CL) reaction and the OTA aptamer conjugated with TEX615 (Park et al., 2013).

A novel fluorescent nucleobase analog, 8-thiophene-2′-deoxyguanosine (ThdG), was inserted into the OTA aptamer to determine the effect of the probe on G4 folding and OTA binding affinity. Interestingly, the ThdG-modified aptamer sites can provide a fluorescent response after OTA binding (Fadock and Manderville, 2017). TiO_2_ quenches non-covalent adsorption of FAM-labeled aptamers TiO_2_ (Sharma et al., 2015). When OTA interacts with its aptamer, the interaction between the FAM-labeled aptamer and TiO_2_ is weakened, resulting in fluorescence recovery.

Based on Tb^3+^, the G4 aptamer and magnetic beads (MBs), a fluorescent aptasensor was reported (Zhang et al., 2013). The OTA aptamer was initially labeled with biotin and conjugated to streptavidin-labeled MBs. Two single-stranded signal probes were added to hybridize with the OTA aptamer to form a duplex. However, when OTA is present, the OTA-aptamer complex is formed and the two single-stranded signal probes are released. After magnetic separation, the liberated single-stranded signal probes significantly increase the fluorescent intensity of Tb^3+^. This sensor could detect OTA as low as 20 pg/mL with high specificity.

A simple and sensitive aptamer/SWCNH analysis for OTA detection was also described (Lv et al., 2017). SWCNH protects DNA from DNase I cleavage. However, binding of OTA to its aptamer results in their detachment from SWCNHs, allowing them to be cleaved and triggering the next cycle. The signal amplification mechanism reduced the LOD for OTA analysis by nearly 20 times.

In Zeng et al. (2019), a labeled aptamer was combined with thioflavin T (ThT) to achieve ratiometric fluorescence resonance energy transfer (FRET) detection. This assay recorded a 0.38 ng/mL LOD for OTA and could complete the assay within 30 s.

The detection tool of OTA in living cells is unexplored. Deng, Wu and co-workers recently reported conformation-resolved aptamer probes to achieve living cell detection of OTA (Xia et al., 2020).

A summary of labeled G4-based fluorescence OTA detection methods is presented in Table 1.

**Table 1:** Labeled G4-based fluorescence OTA detection methods.

**Aptamer (5′-3′)**	**Probe/Quencher**	**LOD**	**References**
d(GATCGGGTGTGGGTGGCGTAAAGGGAGCATCGGACA-FAM)	Single-walled carbon nanohorn	17.2 nM	Lv et al., 2016
	Single-walled carbon nanotubes	24.1 nM	Guo et al., 2011
	Mg^2+^-dependent DNAzyme	140 pmol/L	Guo et al., 2013
d(FAM-GATCGGGTGTGGGTGGCGTAAAGGGAGCATCGGACA)	Nano-graphite	20 nM	Wei et al., 2015
d(TEX615-GATCGGGTGTGGGTGGCGTAAAGGGAGCATCGGACA)	Multi-walled carbon nanotubes	3.9 nM	Park et al., 2013
d(GATCGGGTGTGGGTGGCGTAAAGGGAGCATCGGACA-FAM)	TiO_2_	1.5 nM	Sharma et al., 2015
d(GATCGGGTGTGGGTGGCGTAAAGGGAGCATCGGACA-C6-biotin)	Probe1: d(CCACAC CCGATC); Probe2: d(TGTCCGATGCTC)	20 pg/mL	Zhang et al., 2013
d(FAM-GATCGGGTGTGGGTGGCGTAAAGGGAGCATCGGACA)	Single-walled carbon nanohorn	9.8 nM	Lv et al., 2017

### Label-Free Luminescence OTA Detection Methods

**Figure 4:**
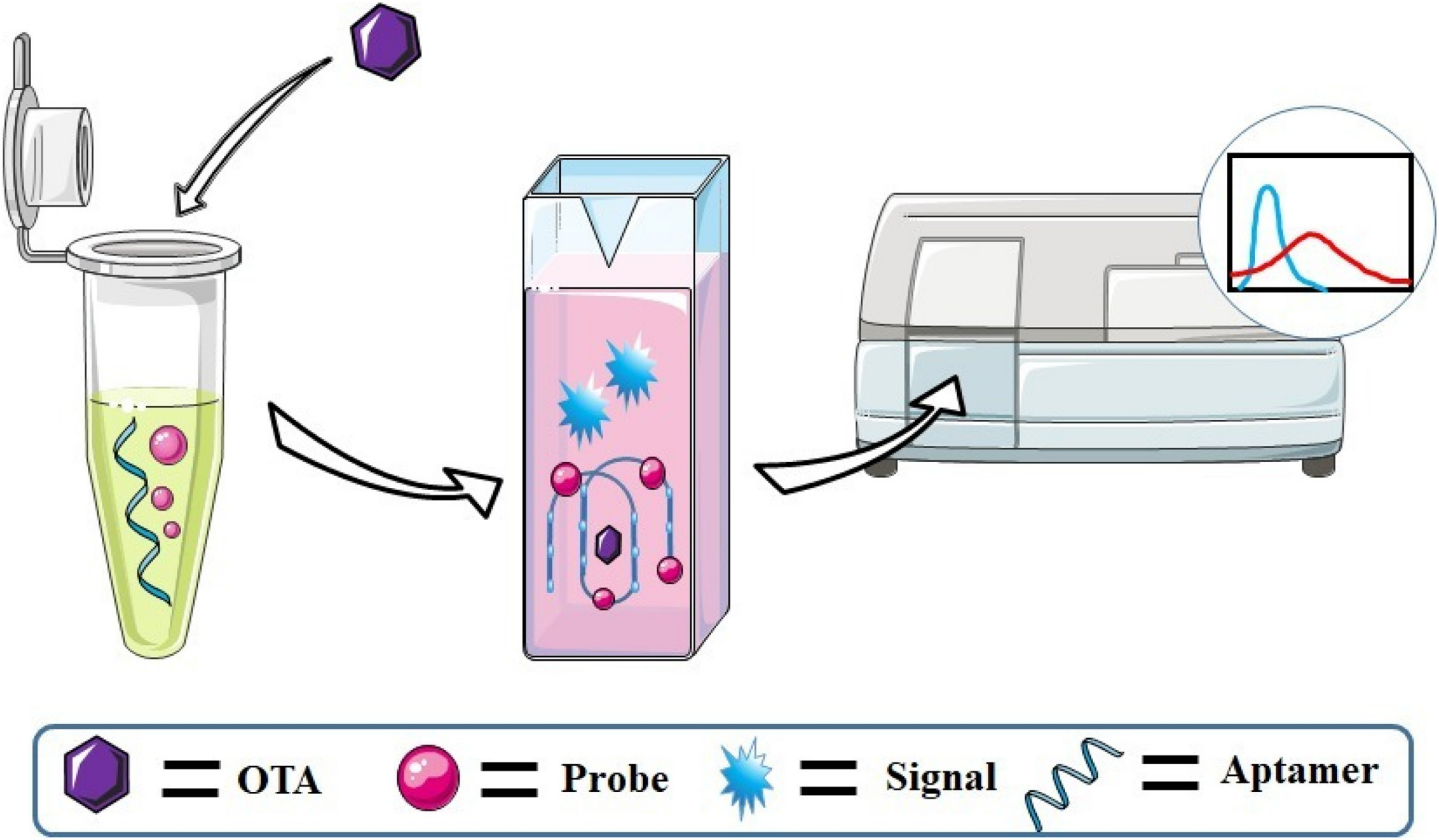
An illustration of the OTA detection process based on G4s formed by label-free aptamer.

While fluorescence labeling is a popular strategy for OTA detection, it has some drawbacks. The labeling of oligonucleotides can be expensive, and the attachment can also interfere with the aptamer-ligand interaction. In contrast, label-free methods are simpler and also lower in cost. Luminescent probes that can recognize the G4 structure formed when the OTA aptamer binds to OTA can be used to develop label-free G4-based OTA assays (Figure 4).

Liu et al. (2019) developed a label-free detection method for OTA using the G4 probe TMPyP, which quenches the fluorescence of CdTe quantum dots (QDs). In the presence of OTA, the TMPyP is sequestered by the aptamer G4, which restores the fluorescence of the CdTe QDs. The detection limit of the designed aptasensor for OTA was 0.16 ng/mL, with a linear range of 0.2–20 ng/mL. Yang et al. utilized aggregation-induced emission (AIE) to develop an OTA detection method (Lv et al., 2019). In the absence of OTA, the aptamer is digested by Exo I and the AIE probe 4,4-(1E,1E)-2,2-(anthracene-9,10-diyl)bis(ethene-2,1-diyl)bis(N,N,N-trimethylbenzenammonium iodide) (DSAI) shows low luminescence. However, when OTA is present, the aptamer adopts a G4 configuration which resists digestion by Exo I. Then, DSAI will aggregate on the surface of the aptamer/OTA complex and produce a strong emission. This probe showed a linear range of 5–500 ng/ml OTA and a detection limit of 1.9 ng/ml.

A G4-based platform has been developed for time-resolved monitoring of OTA (Zhang J.-T. et al., 2019). This simple platform shows good sensitivity to OTA by steady-state emission spectroscopy with a detection limit of 40 nM. It is worth noting that the detection limit shown by the platform through time-resolved emission spectroscopy (TRES) is 10.8 nM, which is about 4 times higher than the sensitivity of the steady state mode.

A simple platform for detecting OTA was reported based on the OTA aptamer and the G4-specific probe zinc (II)-protoporphyrin IX probe. The simple method was sensitive toward OTA with an LOD of 0.03 nM and also displayed good specificity for OTA over other mycotoxins (Liu F. et al., 2018).

A rolling circle amplification (RCA)-activated multisite-catalytic hairpin assembly was used to develop an ultrasensitive fluorescent biosensor for OTA (Wang J. et al., 2019). In this work system, two specially-designed probes, termed Complex I and II, were prepared by combining a circular template with an OTA aptamer, a substrate probe and hairpin probe 1. The presence of the target initiates multiple rounds of RCA, forming numerous Y-DNTs Y-shaped DNA nanotorches that contain a G4 structure for binding to the fluorescent probe, N-methylmesoporphyrin IX. The biosensor was quick to use and showed a very low LOD of 0.0002 ng/mL for OTA.

In a recent study, an OTA detection method based on the amplification of non-enzyme hybridization chain reaction was described (Qian et al., 2020). In the presence of OTA, the fluorescent signals are significantly enhanced due to the interaction of G4 with N-methylmesoporphyrin IX. The LOD for OTA was 4.9 pM.

A fluorescence aptamer-based assay was developed for detecting OTA using ThT (Wu et al., 2018). In the absence of OTA, the OTA aptamers can form G4 structures with ThT dyes, resulting in increased fluorescence. However, OTA displaces ThT from the OTA aptamer resulting in a turn-off signal response.

A ratiometric FRET aptasensor between [Ru(bpy)_3_]^2+^ and silica quantum dots (silica QDs) assaying for testing OTA was reported by Zhu et al. (2020). The OTA aptamer was used to shield [Ru(bpy)_3_]^2+^ into silica nanoparticles. In the presence of OTA, [Ru(bpy)_3_]^2+^ is released and is absorbed onto negatively charged silica QDs, resulting in energy transfer with a 0.08 ng/mL LOD.

A simple detection method based on the unique antiparallel G4 structure formed by OTA was reported (Armstrong-Price et al., 2020). The “turn-on” fluorescence self-signal (λ_ex_/λ_em_ = 256/425 nm) is generated by π-stacking interactions that lead to G4-to-toxin energy transfer (ET). This platform showed an LOD for OTA of ~2 ng/mL.

According to Deore et al. (2019), a coumarin–hemicyanine hybrid (BtC), 4-quinolinium-indole (4QI), and ThT could serve as probes for the OTA aptamer. The dye affinity order was 4QI > BtC > ThT. The LOD of 4QI and BtC for OTA was 6 ng/mL, while the LOD of ThT for OTA was 29 ng/mL.

The hemin-G4 system has activity similar to horse radish peroxidase (HRP), and so can catalyze the production of chemiluminescence signals from luminol in the presence of H_2_O_2_. A chemiluminescence resonance energy transfer aptamer sensor was developed by labeling the OTA aptamer with dabcyl. When OTA is added, chemiluminescence resonance energy transfer occurs luminol (donor) and dabcyl (acceptor). By this means, the limit of OTA detection was 0.22 ng/mL (Jo et al., 2016).

Another hemin-G4 aptamer chemiluminescence assay platform was developed for detecting OTA (Shen et al., 2017). This assay used single silica photonic crystal microspheres, and the oxidation of ABTS^2−^ or luminol by H_2_O_2_ produces chemiluminescence.

Wang Y. et al. (2019) described a label-free and signal-on chemiluminescence strategy with a low LOD for OTA of 0.07 ng/mL. They used a signal probe (triplex-forming oligonucleotide) to catalyze H_2_O_2_ by hemin-G4 unique structure producing a chemiluminescence signal after the specific aptamer captured OTA.

A summary of label-free G4-based luminescence detection methods for OTA is presented in Table 2.

**Table 2:** Label-free G4-based fluorescence OTA detection methods.

**Aptamer (5′-3′)**	**Probe**	**LOD**	**References**
d(GATCGGGTGTGGGTGGCGTAAAGGGAGCATCGGACA)	TMPyP	0.16 ng/mL	Liu et al., 2019
d(GATCGGGTGTGGGTGGCGTAAAGGGAGCATCGGACA)	DSAI	1.9 ng/mL	Lv et al., 2019
d(TGGGTAGGGCGGGTTGGGAAAGATCGGGTGTGGGTGGCGTAAAGGGAGCATCGGACA)	/	4 nM	Yang et al., 2013
d(GATCGGGTGTGGGTGGCGTAAAGGGAGCATCGGACA)	Iridium complex	10.8 nM	Zhang J.-T. et al., 2019
d(GATCGGGTGTGGGTGGCGTAAAGGGAGCATCGGACA)	Protoporphyrin IX zinc(II)	0.03 nM	Liu F. et al., 2018
d(GATCGGGTGTGGGTGGCGTAAAGGGAGCATCGGACATTTTTT)	N-methylmesoporphyrin IX	0.0002 ng/mL	Wang J. et al., 2019
d(GATCGGGTGTGGGTGGCGTAAAGGGAGCATCGGACA)	Thioflavin T	0.4 ng/mL	Wu et al., 2018
d(GATCGGGTGTGGGTGGCGTAAAGGGAGCATCGGACA)	/	2 ng/mL	Armstrong-Price et al., 2020
d(CCGCCCGATCGGGTGTGGGTGGCGTAAAGGGAGCATCGGACACCCGCC)	d(GGGTAGGGCGGGTTGGG)	0.07 ng/mL	Wang Y. et al., 2019
d(GATCGGGTGTGGGTGGCGTAAAGGGAGCATC)	Coumarin–hemicyanine hybrid and 4-quinolinium-indole	6 ng/mL	Deore et al., 2019
	Thioflavin T	29 ng/mL	
d(GATCGGGTGTGGGTGGCGTAAAGGGAGCATCGGACA)	Thioflavin T	0.38 ng/mL	Zeng et al., 2019
d(GATCGGGTGTGGGTGGCGTAAAGGGAGCATCGGACACGCCACCCACAC)	Hairpin 1 and 2	4.9 Pm	Qian et al., 2020

### Colorimetric OTA Detection Methods

**Figure 5:**
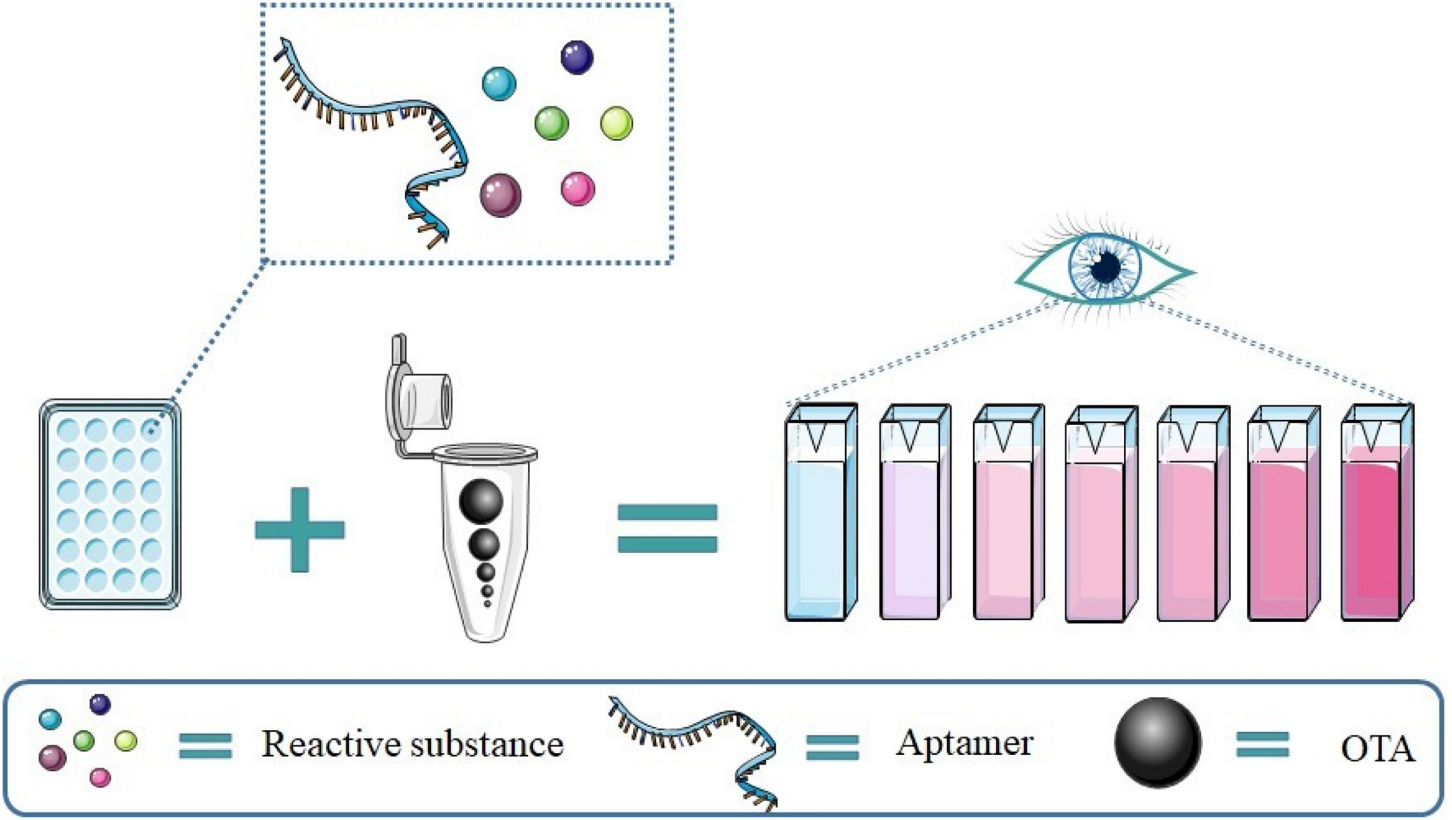
An illustration of a colorimetric OTA detection assay based on the aptamer G4.

Compared to fluorescence signal detection, colorimetric results are easy to read and require simpler equipment, as showed in Figure 5.

5,5′-tetramethylbenzidine (TMB) can be readily oxidized by H_2_O_2_ thus causing the color to change from colorless to blue. This OTA detection platform achieved an LOD of 0.25 nM (Yang et al., 2012).

A label-free colorimetric aptasensor based on a DNAzyme–aptamer conjugate was reported for the detection of OTA in wine (Yang et al., 2013). In this system, the oligonucleotide N1 containing both the OTA-binding aptamer sequence and the horseradish peroxidase (HRP)-mimicking DNAzyme sequence is initially hybridized to a blocking oligonucleotide. When DNA is added, the HRP sequence is liberated due to the formation of the OTA/aptamer complex, leading to a colorimetric response that is with OTA concentration up to 30 nM and with an LOD of 4 nM.

A liposome-based colorimetric assay was subsequently developed using TMB as the substrate (Lin et al., 2018). When OTA is added to the aptamer, the detection probes and HRP-encapsulated liposomes are released. The liberated HRP can catalyze the oxidation of TMB mediated by the H_2_O_2_ oxidation. The absorption intensity at 652 nm increased with OTA concentration from 0.05 to 2.0 ng/mL, and the LOD was 0.023 ng/mL.

A different colorimetric substrate, ABTS, was used by Lee et al. (2014) to detect OTA. In their design, in the presence of OTA, the stem of a hairpin is loosened to freely form a G-quadruplex with covalently conjugated hemin and expresses horse radish peroxidase activity (with the detection limit of several nM). The color of ABST change to green could be observed by the naked eye with an LOD of around 1 nM.

Another colorimetric strategy for detecting OTA used gold nanoparticles (AuNPs). When the OTA aptamer adopts a rigid G4 structure in the presence of OTA, the AuNPs are not protected against salt-induced aggregation thus generating a color change from red to blue (Yang et al., 2011).

Another OTA assay was developed based on the OTA aptamer and the enzyme-induced metallization of gold nanorods (Tian et al., 2020). This assay achieved an result LOD of 0.9 nM.

In another strategy, a DNA sequence was designed that contains the heme aptamer as well as the OTA aptamer (AG4-OTA) (Yu et al., 2018). OTA combines with AG4-OTA to form an antiparallel G4 that resists digestion by Exo I. Meanwhile, the heme aptamer sequence retained in this way can be combines with heme to form a G4-heme DNase that has peroxidase-like activity and can catalyze the oxidation of TMB by H_2_O_2_. The resulting diimine derivative (TMB^2+^). TMB^2+^ can etch AuNRs by oxidizing Au(0) to Au(I), generating a rainbow-like spectrum and providing a multi-color sensor for the visual monitoring of OTA.

A promising on-site visual semi-quantitative detection method for OTA in foods was reported by Tian et al. (2020). This high-resolution colorimetric assay was based on the ability of G4 structures to capture OTA in a matrix, leading to a color change that was visible to the naked eye. The LOD of this method was 9.0 nM.

A list of colorimetric G4-based OTA detection methods are presented in Table 3.

**Table 3:** Colorimetric G4-based OTA detection methods.

**Aptamer (5′-3′)**	**Chromogenic substrate**	**LOD**	**References**
d(GATCGGGTGTGGGTGGCGTAAAGGGAGCATCGGACA)	3,3′,5,5′-tetramethylbenzidine	0.023ng/mL	Lin et al., 2018
d(CTGGGAGGGAGGGAGGGAAAAGATCGGGTGTGGGTGGCGTAAAGGGAGCATCGGACACCCGATCTTTTCCCTCCC)	ABTS	1 nM	Lee et al., 2014
d(GATCGGGTGTGGGTGGCGTAAAGGGAGCATCGGACA-)	Gold nanoparticle	20 nM	Yang et al., 2011
d(CTGGGAGGGAGGGAGGGATCGGGTGTGGGTGGCGTAAAGGGAGCATCGGACACCCGATCCC)	3,3′,5,5′-tetramethylbenzidine	2.5 nM	Yang et al., 2012
d(biotin-AAAAAAAAAAAAGATCGGGTGTGGGTGGCGTAAAGGGAGCATCGG ACA)	Gold nanorods	0.9 nM	Tian et al., 2020
d(-NH2-C6-TGGGTAGGGCGGGTTGGGAAAGATCGGGTGTGGGTGGCGTAAAGGGAGCATCGGACA)	ABTS	4.28 pg/mL	Shen et al., 2017

### Other OTA Detection Methods

Gu et al. (2019) developed an electrochemical OTA detection method using AuNP@CuCoPBA, which possesses good electrochemical conductivity, high aptamer affinity, and strong G4 stability. Using electrochemical impedance spectroscopy, this platform achieved an LOD of 5.2 fg/mL for OTA.

A versatile photoelectrochemical (PEC) immunosensor was reported for the simultaneous detection of multiple ochratoxins (Qileng et al., 2018). This assay was based on the direct growth of CdS nanorods on Fluorine-doped Tin Oxide (FTO) as the photoelectrode and Au nanoflowers-modified glass carbon electrode (GCE) as the bioelectrode. G4 plays catalyzes the oxidation of 4-chloro-1-naphthol (4-CN) by H_2_O_2_ to yield biocatalytic precipitation (BCP) on the bioelectrode, followed by Cu^2+^ release when the bioelectrode is treated with moderate acid. This leads to a decrease of the photocurrent of the photoelectrode by the formation of CuS.

Another OTA detection assay employed localized surface plasmon resonance (LSPR). The magnitude of the LSPR wavelength change depends on the changes in the analyte and refractive index of the two positions relative to the surface of the nanoparticle. In this system, the addition of OTA causes the aptamer to adopt a G4 structure, leading to a shift of the LSPR peak associated with the local refractive index change near the gold nanorod surface. The lower limit of the system for OTA detection was below 1 nM (Park et al., 2014).

## Conclusions

In the review, we have reviewed the latest developments in G4-based detection platforms for OTA in recent years. These assays are generally based on the ability of the OTA aptamer to adopt a G4 configuration when it binds to OTA. Because of its high affinity and selectivity or OTA, the aptamer can be used to construct various G4-based platforms for OTA detection. These assays have the advantages of good sensitivity, high specificity, and low cost of nucleic acid aptamers. Many studies described in this review have exploited the change of luminescence before and after the formation of the G4 structure. Various OTA detection systems have been proposed based on G4 topology transformation. The OTA aptamer can target OTA with high affinity an selectivity even in a complex matrix, which could reduce the cost and increase the robustness of food safety testing. Compare to conventional instrument testing, G4-based luminescence OTA detection assays are promising due to their convenience and sensitivity, as well as the ability to perform rapid, low-cost, and real-time assays.

These assays can be further divided into “labeled” and “label-free” methods. The advantages of label-free techniques is that it avoids the expensive and time-consuming process of conjugating dyes to nucleic acids, which may also affect its affinity for OTA and perturb the sensing mechanism.

Given that OTA is found in foodstuffs, it is necessary to consider possible signal interference caused by the complexity of the sample components. One approach that deserves further attention is the use of metal complexes as the G4 probes luminescence detection. Metal complexes display large Stokes shifts, high quantum yields, and lengthy phosphorescence lifetimes, which can allow their emission to be distinguished from interfering fluorophores commonly found in environmental and biological media using time-resolved emission spectroscopy. This could increase the speed of detection by reducing the sample preparation steps.

Besides luminescence detection, colorimetric assays have also received widespread attention because their final output visible to the naked eye. However, colorimetric assays usually require multiple steps (e.g., enzymatic reaction) or multiple components (e.g., nanoparticles) to effect the color change. Moreover, colorimetric outputs less robust to interference compared to luminescence outputs. These are challenges that have to be overcome to advance colorimetric methods for OTA detection in real life. Finally, electrochemistry biosensors have the advantages, high efficiency and recyclability and sensitivity. However, the initial setup of the platform may be costly and electrode maintenance needs to be considered as well.

As these *in vitro* methods progress in sensitivity and selectivity, we hope that in the future, more effective *in vivo* detection strategies can be developed for sensing OTA in both animal and non-animal products.

## Author Contributions

The authors contributed to the data preparation, drafted, and revised the manuscript. All authors read and approved the final manuscript.

## Conflict of Interest

The authors declare that the research was conducted in the absence of any commercial or financial relationships that could be construed as a potential conflict of interest.

## References

[B1] Abarca M. L. Accensi F. Bragulat M. R. Cabanes F. J. (2001). Current importance of ochratoxin A-producing *Aspergillus* spp. J. Food Prot. 64, 903–906. 10.4315/0362-028X-64.6.903 11403149

[B2] Al-Anati L. Petzinger E. (2006). Immunotoxic activity of ochratoxin A. J. Vet. Pharmacol. Ther. 29, 79–90. 10.1111/j.1365-2885.2006.00718.x 16515661

[B3] Armstrong-Price D. E. Deore P. S. Manderville R. A. (2020). Intrinsic “turn-on” aptasensor detection of ochratoxin A using energy-transfer fluorescence. J. Agric. Food Chem. 68, 2249–2255. 10.1021/acs.jafc.9b07391 31986034

[B4] Bing T. Zheng W. Zhang X. Shen L. Liu X. Wang F.. (2017). Triplex-quadruplex structural scaffold: a new binding structure of aptamer. Sci. Rep. 7:15467. 10.1038/s41598-017-15797-5 29133961 PMC5684193

[B5] Blank M. Blind M. (2005). Aptamers as tools for target validation. Curr. Opin. Chem. Biol. 9, 336–342. 10.1016/j.cbpa.2005.06.011 16006181

[B6] Burge S. Parkinson G. N. Hazel P. Todd A. K. Neidle S. (2006). Quadruplex DNA: sequence, topology and structure. Nucleic Acids Res. 34, 5402–5415. 10.1093/nar/gkl655 17012276 PMC1636468

[B7] Chompurat S. Sato K. Mahakarnchanakul W. Sirison J. Kladpan S. (2007). Determination of ochratoxin A in roasted coffee and corn by immunoaffinity column cleanup with high-performance thin layer chromatography. JSM Mycotoxins 2006, 197–201. 10.2520/myco1975.2006.Suppl4_197

[B8] Cogoi S. Xodo L. E. (2006). G-quadruplex formation within the promoter of the KRAS proto-oncogene and its effect on transcription. Nucleic Acids Res. 34, 2536–2549. 10.1093/nar/gkl286 16687659 PMC1459413

[B9] Creppy E. E. Schlegel M. Roschenthaler R. Dirheimer G. (1980). Phenylalanine prevents acute poisoning by ochratoxina in mice. Toxicol. Lett. 6, 77–80. 10.1016/0378-4274(80)90171-X 7414623

[B10] De Armond R. Wood S. Sun D. Hurley L. H. Ebbinghaus S. W. (2005). Evidence for the presence of a guanine quadruplex forming region within a polypurine tract of the hypoxia inducible factor 1α promoter. Biochemistry 44, 16341–16350. 10.1021/bi051618u 16331995

[B11] De Cian A. Lacroix L. Douarre C. Temime-Smaali N. Trentesaux C. Riou J.-F.. (2008). Targeting telomeres and telomerase. Biochimie 90, 131–155. 10.1016/j.biochi.2007.07.011 17822826

[B12] Deore P. S. Gray M. D. Chung A. J. Manderville R. A. (2019). Ligand-induced G-quadruplex polymorphism: a DNA nanodevice for label-free aptasensor platforms. J. Am. Chem. Soc. 141, 14288–14297. 10.1021/jacs.9b06533 31436972

[B13] Dong Z.-Z. Lu L. Wang W. Li G. Kang T.-S. Han Q. (2017). Luminescent detection of nicking endonuclease Nb. BsmI activity by using a G-quadruplex-selective iridium (III) complex in aqueous solution. Sens. Actuators B Chem. 246, 826–832. 10.1016/j.snb.2017.02.156

[B14] El Khoury A. Atoui A. (2010). Ochratoxin a: general overview and actual molecular status. Toxins 2, 461–493. 10.3390/toxins2040461 22069596 PMC3153212

[B15] Ellington A. D. Szostak J. W. (1992). Selection *in vitro* of single-stranded DNA molecules that fold into specific ligand-binding structures. Nature 355:850. 10.1038/355850a0 1538766

[B16] Fadock K. L. Manderville R. A. (2017). DNA aptamer–target binding motif revealed using a fluorescent guanine probe: implications for food toxin detection. ACS omega 2, 4955–4963. 10.1021/acsomega.7b00782 30023732 PMC6044742

[B17] Filek G. Lindner W. (1996). Determination of the mycotoxin moniliformin in cereals by high-performance liquid chromatography and fluorescence detection. J. Chromatogr. A 732, 291–298. 10.1016/0021-9673(95)01275-3

[B18] Frenich A. G. Romero-González R. Gómez-Pérez M. L. Vidal J. L. M. (2011). Multi-mycotoxin analysis in eggs using a QuEChERS-based extraction procedure and ultra-high-pressure liquid chromatography coupled to triple quadrupole mass spectrometry. J. Chromatogr. A 1218, 4349–4356. 10.1016/j.chroma.2011.05.005 21621786

[B19] Frohlich A. Marquardt R. Bernatsky A. (1988). Quantitation of ochratoxin A: use of reverse phase thin-layer chromatography for sample cleanup followed by liquid chromatography or direct fluorescence measurement. J. Assoc. Off. Anal. Chem. 71, 949–953. 10.1093/jaoac/71.5.949 3235415

[B20] Georgiades S. N. Abd Karim N. H. Suntharalingam K. Vilar R. (2010). Interaction of metal complexes with G-quadruplex DNA. Angew. Chem. Int. 49, 4020–4034. 10.1002/anie.200906363 20503216

[B21] Gu C. Yang L. Wang M. Zhou N. He L. Zhang Z.. (2019). A bimetallic (Cu-Co) Prussian Blue analogue loaded with gold nanoparticles for impedimetric aptasensing of ochratoxin A. Microchim. Acta 186:343. 10.1007/s00604-019-3479-5 31076934

[B22] Guo Z. Ren J. Wang J. Wang E. (2011). Single-walled carbon nanotubes based quenching of free FAM-aptamer for selective determination of ochratoxin A. Talanta 85, 2517–2521. 10.1016/j.talanta.2011.08.015 21962677

[B23] Guo Z. Wang J. Wang E. (2013). Signal-amplification detection of small molecules by use of Mg^2+^-dependent DNAzyme. Anal. Bioanal. Chem. 405, 4051–4057. 10.1007/s00216-013-6788-2 23407810

[B24] Huang L. C. Zheng N. Zheng B. Q. Wen F. Cheng J. B. Han R. W.. (2014). Simultaneous determination of aflatoxin M1, ochratoxin A, zearalenone and alpha-zearalenol in milk by UHPLC-MS/MS. Food Chem. 146, 242–249. 10.1016/j.foodchem.2013.09.047 24176338

[B25] Huppert J. L. (2008). Four-stranded nucleic acids: structure, function and targeting of G-quadruplexes. Chem. Soc. Rev. 37, 1375–1384. 10.1039/b702491f 18568163

[B26] Huppert J. L. Balasubramanian S. (2005). Prevalence of quadruplexes in the human genome. Nucleic Acids Res. 33, 2908–2916. 10.1093/nar/gki609 15914667 PMC1140081

[B27] Jiao Y. Blaas W. Rühl C. Weber R. (1992). Identification of ochratoxin A in food samples by chemical derivatization and gas chromatography-mass spectrometry. J. Chromatogr. A 595, 364–367. 10.1016/0021-9673(92)85183-T 1577913

[B28] Jo E.-J. Mun H. Kim S.-J. Shim W.-B. Kim M.-G. (2016). Detection of ochratoxin A (OTA) in coffee using chemiluminescence resonance energy transfer (CRET) aptasensor. Food Chem. 194, 1102–1107. 10.1016/j.foodchem.2015.07.152 26471659

[B29] Kupski L. Badiale-Furlong E. (2015). Principal components analysis: an innovative approach to establish interferences in ochratoxin A detection. Food Chem. 177, 354–360. 10.1016/j.foodchem.2015.01.005 25660897

[B30] Kwok C. K. Merrick C. J. (2017). G-Quadruplexes: prediction, characterization, and biological application. Trends Biotechnol. 35, 997–1013. 10.1016/j.tibtech.2017.06.012 28755976

[B31] Lee J. Jeon C. H. Ahn S. J. Ha T. H. (2014). Highly stable colorimetric aptamer sensors for detection of ochratoxin A through optimizing the sequence with the covalent conjugation of hemin. Analyst 139, 1622–1627. 10.1039/C3AN01639K 24519363

[B32] Li X.-M. Zheng K.-W. Zhang J.-Y. Liu H.-H. Yuan B.-F. Hao Y.-H.. (2015). Guanine-vacancy–bearing G-quadruplexes responsive to guanine derivatives. Proc. Natl. Acad. Sci. U.S.A. 112, 14581–14586. 10.1073/pnas.1516925112 26553979 PMC4664295

[B33] Lim E. Pon A. Djoumbou Y. Knox C. Shrivastava S. Guo A. C.. (2009). T3DB: a comprehensively annotated database of common toxins and their targets. Nucleic Acids Res. 38, 781–786. 10.1093/nar/gkp934 19897546 PMC2808899

[B34] Lin C. Zheng H. Sun M. Guo Y. Luo F. Guo L.. (2018). Highly sensitive colorimetric aptasensor for ochratoxin A detection based on enzyme-encapsulated liposome. Anal. Chim. Acta 1002, 90–96. 10.1016/j.aca.2017.11.061 29306417

[B35] Lin S. Kang T.-S. Lu L. Wang W. Ma D.-L. Leung C.-H. (2016a). A G-quadruplex-selective luminescent probe with an anchor tail for the switch-on detection of thymine DNA glycosylase activity. Biosens. Bioelectron. 86, 849–857. 10.1016/j.bios.2016.07.082 27494808

[B36] Lin S. Lu L. Kang T.-S. Mergny J.-L. Leung C.-H. Ma D.-L. (2016b). Interaction of an iridium (III) complex with G-quadruplex DNA and its application in luminescent switch-on detection of siglec-5. Anal. Chem. 88, 10290–10295. 10.1021/acs.analchem.6b03128 27678199

[B37] Lin S. Wang W. Hu C. Yang G. Ko C.-N. Ren K.. (2017). The application of a G-quadruplex based assay with an iridium (iii) complex to arsenic ion detection and its utilization in a microfluidic chip. J. Mater. Chem. B 5, 479–484. 10.1039/C6TB02656G 32263664

[B38] Lipps H. J. Rhodes D. (2009). G-quadruplex structures: *in vivo* evidence and function. Trends Cell Biol. 19, 414–422. 10.1016/j.tcb.2009.05.002 19589679

[B39] Liu F. Ding A. Zheng J. Chen J. Wang B. (2018). A label-free aptasensor for ochratoxin A detection based on the structure switch of aptamer. Sensors 18:1769. 10.3390/s18061769 29857594 PMC6022100

[B40] Liu L. Tanveer Z. I. Jiang K. Huang Q. Zhang J. Wu Y.. (2019). Label-free fluorescent aptasensor for ochratoxin-A detection based on CdTe quantum dots and (N-Methyl-4-pyridyl) porphyrin. Toxins 11:447. 10.3390/toxins11080447 31357671 PMC6724026

[B41] Liu X. Liu X. Huang P. Wei F. Ying G. Lu J.. (2018). Regeneration and reuse of immunoaffinity column for highly efficient clean-up and economic detection of Ochratoxin A in malt and ginger. Toxins 10:462. 10.3390/toxins10110462 30413078 PMC6266469

[B42] Lu L. Mao Z. Kang T.-S. Leung C.-H. Ma D.-L. (2016a). A versatile nanomachine for the sensitive detection of platelet-derived growth factor-BB utilizing a G-quadruplex-selective iridium (III) complex. Biosens. Bioelectron. 85, 300–309. 10.1016/j.bios.2016.05.026 27183280

[B43] Lu L. Wang M. Mao Z. Kang T.-S. Chen X.-P. Lu J.-J.. (2016b). A novel dinuclear iridium (III) complex as a G-quadruplex-selective probe for the luminescent switch-on detection of transcription factor HIF-1α. Sci. Rep. 6:22458. 10.1038/srep22458 26932240 PMC4773817

[B44] Lu L. Wang W. Wang M. Kang T.-S. Lu J.-J. Chen X.-P.. (2016c). A luminescent G-quadruplex-selective iridium (III) complex for the label-free detection of lysozyme. J. Mater. Chem. B 4, 2407–2411. 10.1039/C6TB00426A 32263190

[B45] Lu L. Wang W. Yang C. Kang T.-S. Leung C.-H. Ma D.-L. (2016d). Iridium (iii) complexes with 1, 10-phenanthroline-based N∧ N ligands as highly selective luminescent G-quadruplex probes and application for switch-on ribonuclease H detection. J. Mater. Chem. B 4, 6791–6796. 10.1039/C6TB02316A 32263573

[B46] Lv L. Cui C. Liang C. Quan W. Wang S. Guo Z. (2016). Aptamer-based single-walled carbon nanohorn sensors for ochratoxin A detection. Food Control 60, 296–301. 10.1016/j.foodcont.2015.08.002

[B47] Lv L. Cui C. Xie W. Sun W. Ji S. Tian J.. (2019). A label-free aptasensor for turn-on fluorescent detection of ochratoxin A based on aggregation-induced emission probe. Methods Appl. Fluoresc. 8:015003. 10.1088/2050-6120/ab4edf 31622960

[B48] Lv L. Li D. Cui C. Zhao Y. Guo Z. (2017). Nuclease-aided target recycling signal amplification strategy for ochratoxin A monitoring. Biosens. Bioelectron. 87, 136–141. 10.1016/j.bios.2016.08.024 27542086

[B49] Ma D.-L. Wang W. Mao Z. Yang C. Chen X.-P. Lu J.-J.. (2016). A tutorial review for employing enzymes for the construction of G-quadruplex-based sensing platforms. Anal. Chim. Acta 913, 41–54. 10.1016/j.aca.2016.01.043 26944988

[B50] Ma D.-L. Zhang Z. Wang M. Lu L. Zhong H.-J. Leung C.-H. (2015). Recent developments in G-quadruplex probes. Chem. Biol. 22, 812–828. 10.1016/j.chembiol.2015.06.016 26190823

[B51] Ma D. L. Wu C. Dong Z. Z. Tam W. S. Wong S. W. Yang C. (2017). The development of G-quadruplex-based assays for the detection of small molecules and toxic substances. Chem. Asian J. 12, 1851–1860. 10.1002/asia.201700533 28470784

[B52] McKeague M. Velu R. Hill K. Bardoczy V. Meszaros T. Derosa M. C. (2014). Selection and characterization of a novel DNA aptamer for label-free fluorescence biosensing of ochratoxin A. Toxins 6, 2435–2452. 10.3390/toxins6082435 25153252 PMC4147592

[B53] Mishra R. K. Hayat A. Catanante G. Istamboulie G. Marty J. L. (2016). Sensitive quantitation of Ochratoxin A in cocoa beans using differential pulse voltammetry based aptasensor. Food Chem. 192, 799–804. 10.1016/j.foodchem.2015.07.080 26304413

[B54] Monchaud D. Teulade-Fichou M.-P. (2008). A hitchhiker's guide to G-quadruplex ligands. Org. Biomol. Chem. 6, 627–636. 10.1039/B714772B 18264563

[B55] Morrissey D. (2003). RNA Interference Mediated Inhibition of Hepatitis B Virus (HBV) Using Short Interfering Nucleic Acid (siNA). U.S. Patent No. 10/244,647. Washington, DC: U.S. Patent and Trademark Office.

[B56] Park J.-H. Byun J.-Y. Mun H. Shim W.-B. Shin Y.-B. Li T.. (2014). A regeneratable, label-free, localized surface plasmon resonance (LSPR) aptasensor for the detection of ochratoxin A. Biosens. Bioelectron. 59, 321–327. 10.1016/j.bios.2014.03.059 24747570

[B57] Park L. Kim J. Lee J. H. (2013). Role of background observed in aptasensor with chemiluminescence detection. Talanta 116, 736–742. 10.1016/j.talanta.2013.07.072 24148468

[B58] Pussemier L. Pierard J. Y. Anselme M. Tangni E. K. Motte J. C. Larondelle Y. (2006). Development and application of analytical methods for the determination of mycotoxins in organic and conventional wheat. Food Addit. Contam. 23, 1208–1218. 10.1080/02652030600699312 17071524

[B59] Qian M. Hu W. Wang L. Wang Y. Dong Y. (2020). A non-enzyme and non-label sensitive fluorescent aptasensor based on simulation-assisted and target-triggered hairpin probe self-assembly for ochratoxin A detection. Toxins 12:376. 10.3390/toxins12060376 32517279 PMC7354513

[B60] Qileng A. Wei J. Lu N. Liu W. Cai Y. Chen M.. (2018). Broad-specificity photoelectrochemical immunoassay for the simultaneous detection of ochratoxin A, ochratoxin B and ochratoxin C. Biosens. Bioelectron. 106, 219–226. 10.1016/j.bios.2018.02.004 29428592

[B61] Raters M. Matissek R. (2008). Thermal stability of aflatoxin B 1 and ochratoxin A. Mycotoxin Res. 24, 130–134. 10.1007/BF03032339 23604747

[B62] Richard J. L. (2007). Some major mycotoxins and their mycotoxicoses-an overview. Int. J. Food Microbiol. 119, 3–10. 10.1016/j.ijfoodmicro.2007.07.019 17719115

[B63] Roland A. Bros P. Bouisseau A. Cavelier F. Schneider R. (2014). Analysis of ochratoxin A in grapes, musts and wines by LC-MS/MS: first comparison of stable isotope dilution assay and diastereomeric dilution assay methods. Anal. Chim. Acta 818, 39–45. 10.1016/j.aca.2014.02.006 24626401

[B64] Santos E. Vargas E. (2002). Immunoaffinity column clean-up and thin layer chromatography for determination of ochratoxin A in green coffee. Food Addit. Contam. 19, 447–458. 10.1080/02652030110213717 12028643

[B65] Sharma A. Hayat A. Mishra R. Catanante G. Bhand S. Marty J. (2015). Titanium dioxide nanoparticles (TiO_2_) quenching based aptasensing platform: application to ochratoxin A detection. Toxins 7, 3771–3784. 10.3390/toxins7093771 26402704 PMC4591649

[B66] Shen P. Li W. Liu Y. Ding Z. Deng Y. Zhu X.. (2017). High-throughput low-background g-quadruplex aptamer chemiluminescence assay for ochratoxin A using a single photonic crystal microsphere. Anal. Chem. 89, 11862–11868. 10.1021/acs.analchem.7b03592 28988477

[B67] Shephard G. S. (2008). Impact of mycotoxins on human health in developing countries. Food Addit. Contam. 25, 146–151. 10.1080/02652030701567442 18286404

[B68] Siddiqui-Jain A. Grand C. L. Bearss D. J. Hurley L. H. (2002). Direct evidence for a G-quadruplex in a promoter region and its targeting with a small molecule to repress c-MYC transcription. Proc. Natl. Acad. Sci. U.S.A. 99, 11593–11598. 10.1073/pnas.182256799 12195017 PMC129314

[B69] Simonsson T. (2001). G-quadruplex DNA structures–variations on a theme. Biol. Chem. 382, 621–628. 10.1515/BC.2001.073 11405224

[B70] Soleas G. J. Yan J. Goldberg D. M. (2001). Assay of ochratoxin A in wine and beer by high-pressure liquid chromatography photodiode array and gas chromatography mass selective detection. J. Agric. Food Chem. 49, 2733–2740. 10.1021/jf0100651 11409959

[B71] Teixeira T. Hoeltz M. Einloft T. Dottori H. Manfroi V. Noll I. (2011). Determination of ochratoxin A in wine from the southern region of Brazil by thin layer chromatography with a charge-coupled detector. Food Addit. Contam. B 4, 289–293. 10.1080/19393210.2011.638088 24786253

[B72] Tian F. Zhou J. Fu R. Cui Y. Zhao Q. Jiao B.. (2020). Multicolor colorimetric detection of ochratoxin A via structure-switching aptamer and enzyme-induced metallization of gold nanorods. Food Chem. 320:126607. 10.1016/j.foodchem.2020.126607 32203832

[B73] Umar S. Arshad A. Ahmad B. Arshad M. (2012). Clinico biochemical and haematological changes in broilers induced by concurrent exposure to aflatoxin B1 and ochratoxin A. J. Public Health Biol. Sci. 1, 79–85. 10.13140/2.1.2854.3681

[B74] Valenta H. (1998). Chromatographic methods for the determination of ochratoxin A in animal and human tissues and fluids. J. Chromatogr. A 815, 75–92. 10.1016/S0021-9673(98)00163-0 9718709

[B75] Varizhuk A. Ischenko D. Tsvetkov V. Novikov R. Kulemin N. Kaluzhny D.. (2017). The expanding repertoire of G4 DNA structures. Biochimie 135, 54–62. 10.1016/j.biochi.2017.01.003 28109719

[B76] Venkataramana M. Rashmi R. Uppalapati S. R. Chandranayaka S. Balakrishna K. Radhika M.. (2015). Development of sandwich dot-ELISA for specific detection of Ochratoxin A and its application on to contaminated cereal grains originating from India. Front. Microbiol. 6:511. 10.3389/fmicb.2015.00511 26074899 PMC4443250

[B77] Wang B. Wu Y. Chen Y. Weng B. Xu L. Li C. (2016). A highly sensitive aptasensor for OTA detection based on hybridization chain reaction and fluorescent perylene probe. Biosens. Bioelectron. 81, 125–130. 10.1016/j.bios.2016.02.062 26938491

[B78] Wang J. Wang Y. Liu S. Wang H. Zhang X. Song X.. (2019). Primer remodeling amplification-activated multisite-catalytic hairpin assembly enabling the concurrent formation of Y-shaped DNA nanotorches for the fluorescence assay of ochratoxin A. Analyst 144, 3389–3397. 10.1039/C9AN00316A 30990481

[B79] Wang M. Wang W. Kang T.-S. Leung C.-H. Ma D.-L. (2015). Development of an Iridium (III) complex as a G-quadruplex probe and its application for the G-quadruplex-based luminescent detection of picomolar insulin. Anal. Chem. 88, 981–987. 10.1021/acs.analchem.5b04064 26607385

[B80] Wang M. Wang W. Liu C. Liu J. Kang T.-S. Leung C.-H. (2017). A luminescence switch-on assay for the detection of specific gene deletion using G-quadruplex DNA and silver nanoclusters. Mater. Chem. Front. 1, 128–131. 10.1039/C6QM00068A

[B81] Wang Q. Chen M. Zhang H. Wen W. Zhang X. Wang S. (2016). Enhanced electrochemiluminescence of RuSi nanoparticles for ultrasensitive detection of ochratoxin A by energy transfer with CdTe quantum dots. Biosens. Bioelectron. 79, 561–567. 10.1016/j.bios.2015.12.098 26749097

[B82] Wang Y. Fang Z. Ning G. Mao S. Wu Y. Wu S. (2019). G-quadruplex-bridged triple-helix aptamer probe strategy: a label-free chemiluminescence biosensor for ochratoxin A. Sens. Actuators B Chem. 298:126867 10.1016/j.snb.2019.126867

[B83] Wei Y. Zhang J. Wang X. Duan Y. (2015). Amplified fluorescent aptasensor through catalytic recycling for highly sensitive detection of ochratoxin A. Biosens. Bioelectron. 65, 16–22. 10.1016/j.bios.2014.09.100 25461133

[B84] Wu K. Ma C. Zhao H. He H. Chen H. (2018). Label-free G-quadruplex aptamer fluorescence assay for Ochratoxin A using a thioflavin T probe. Toxins 10:198. 10.3390/toxins10050198 29757205 PMC5983254

[B85] Wu Y. Brosh R. M. Jr. (2010). G-quadruplex nucleic acids and human disease. FEBS J. 277, 3470–3488. 10.1111/j.1742-4658.2010.07760.x 20670277 PMC2923685

[B86] Xia X. Zhang T. Deng S. Chen J. Wu S. He C. (2020). Visualization of mycotoxins in living cells using conformation-resolved aptamer nanoprobes. ACS Sustain. Chem. Eng. 8, 9920–9925. 10.1021/acssuschemeng.0c03399

[B87] Yang C. Lates V. Prieto-Simón B. Marty J.-L. Yang X. (2013). Rapid high-throughput analysis of ochratoxin A by the self-assembly of DNAzyme–aptamer conjugates in wine. Talanta 116, 520–526. 10.1016/j.talanta.2013.07.011 24148439

[B88] Yang C. Lates V. Prieto-Simon B. Marty J. L. Yang X. (2012). Aptamer-DNAzyme hairpins for biosensing of Ochratoxin A. Biosens. Bioelectron. 32, 208–212. 10.1016/j.bios.2011.12.011 22221796

[B89] Yang C. Wang Y. Marty J.-L. Yang X. (2011). Aptamer-based colorimetric biosensing of Ochratoxin A using unmodified gold nanoparticles indicator. Biosens. Bioelectron. 26, 2724–2727. 10.1016/j.bios.2010.09.032 20970980

[B90] Yang J. Yang Y. C. E. Khan H. F. Xie H. Ringler C. Ogilvie A.. (2018). Quantifying the sustainability of water availability for the water-food-energy-ecosystem Nexus in the Niger River Basin. Earths Future 6, 1292–1310. 10.1029/2018EF000923 31032375 PMC6473704

[B91] Yin X. Wang S. Liu X. He C. Tang Y. Li Q.. (2017). Aptamer-based colorimetric biosensing of Ochratoxin A in fortified white grape wine sample using unmodified gold nanoparticles. Anal. Sci. 33, 659–664. 10.2116/analsci.33.659 28603182

[B92] Yu X. Lin Y. Wang X. Xu L. Wang Z. Fu F. (2018). Exonuclease-assisted multicolor aptasensor for visual detection of ochratoxin A based on G-quadruplex-hemin DNAzyme-mediated etching of gold nanorod. Mikrochim Acta 185:259. 10.1007/s00604-018-2811-9 29680954

[B93] Yuan J. Deng D. Lauren D. R. Aguilar M.-I. Wu Y. (2009). Surface plasmon resonance biosensor for the detection of ochratoxin A in cereals and beverages. Anal. Chim. Acta 656, 63–71. 10.1016/j.aca.2009.10.003 19932816

[B94] Zeng H. Zhu Y. Ma L. Xia X. Li Y. Ren Y. (2019). G-quadruplex specific dye-based ratiometric FRET aptasensor for robust and ultrafast detection of toxin. Dyes Pigm. 164, 35–42. 10.1016/j.dyepig.2019.01.005

[B95] Zhang J. Zhang X. Yang G. Chen J. Wang S. (2013). A signal-on fluorescent aptasensor based on Tb3+ and structure-switching aptamer for label-free detection of ochratoxin A in wheat. Biosens. Bioelectron. 41, 704–709. 10.1016/j.bios.2012.09.053 23089328

[B96] Zhang J.-T. Kang T.-S. Wong S.-Y. Pei R.-J. Ma D.-L. Leung C.-H. (2019). An iridium (III) complex/G-quadruplex ensemble for detection of ochratoxin A based on long-lifetime luminescent. Anal. Biochem. 580, 49–55. 10.1016/j.ab.2019.06.005 31194944

[B97] Zhang X. Li M. Cheng Z. Ma L. Zhao L. Li J. (2019). A comparison of electronic nose and gas chromatography–mass spectrometry on discrimination and prediction of ochratoxin A content in *Aspergillus carbonarius* cultured grape-based medium. Food Chem. 297:124850. 10.1016/j.foodchem.2019.05.124 31253256

[B98] Zhu X. Li W. Lin L. Huang X. Xu H. Yang G.. (2020). Target-responsive ratiometric fluorescent aptasensor for OTA based on energy transfer between [Ru (bpy)_3_]^2+^ and silica quantum dots. Mikrochim Acta 187, 1–8. 10.1007/s00604-020-04245-3 32291531

[B99] Zou X. Chen C. Huang X. Chen X. Wang L. Xiong Y. (2016). Phage-free peptide ELISA for ochratoxin A detection based on biotinylated mimotope as a competing antigen. Talanta 146, 394–400. 10.1016/j.talanta.2015.08.049 26695281

